# The anti-obesity effects of *Clostridium butyricum* B-3 and its impact on gut microbiota

**DOI:** 10.1128/aem.01152-25

**Published:** 2025-10-16

**Authors:** Yue Meng, Lu-Jia Bai, Jun Meng, Chang-He Ding, Jun Xi

**Affiliations:** 1 Food Engineering Technology Research Center/Key Laboratory of Henan Province, Henan University of Technology 47901, Zhengzhou, Henan, China; Universita degli Studi di Napoli Federico II, Portici, Italy

**Keywords:** *Clostridium butyricum*, butyric acid, anti-obesity, gut microbiota

## Abstract

Obesity has emerged as a significant global health threat. In this study, we investigated the anti-obesity properties of *Clostridium butyricum* B-3, a strain noted for its high butyric acid production. This strain was administered to mice on a high-fat diet (HFD) to assess its potential anti-obesity effects. Our findings indicate that three different dosages of *C. butyricum* B-3 significantly (*P* < 0.05) mitigated excessive weight gain and ameliorated kidney and liver damage in HFD mice. A medium dose (10^7^ CFU/day) of *C. butyricum* B-3 markedly reduced levels of total cholesterol, triglycerides and low-density lipoprotein cholesterol, and elevated levels of high-density lipoprotein cholesterol in HFD mice. Additionally, this dosage lowered serum levels of inflammatory cytokines, including IL-6, IL-10, and TNF-α. Furthermore, treatment with *C. butyricum* B-3 enhanced the diversity and relative abundance of gut microbiota in HFD-fed mice. Notably, the gut microbiota composition of the high-dose (10^8^ CFU/day) group closely resembled that of mice fed a normal diet. *C. butyricum* B-3 also significantly increased the concentrations of acetic acid, propionic acid, and butyric acid in the gut. In summary, *C. butyricum* B-3 demonstrated significant anti-obesity effects and the ability to regulate intestinal microbiota in HFD mice.

**IMPORTANCE:**

Obesity represents one of the most severe global health challenges of our time. *Clostridium butyricum* has recently been recognized as a next-generation probiotic with considerable potential for applications in intestinal health, metabolic diseases, and neuroprotection. Our research focused on a specific strain of *C. butyricum* known for its anti-obesity effects, which displayed robust capabilities in mitigating obesity and regulating intestinal flora. This strain holds significant promise for development in anti-obesity nutritional products and pharmaceuticals.

## INTRODUCTION

*Clostridium butyricum* is a Gram-positive spore-forming bacterium that predominantly ferments undigested dietary fibers and carbohydrates in the intestines, primarily producing butyric acid. This microorganism thrives in a variety of environments, predominantly as an obligate anaerobe within the *Clostridium* clusters IV and XIVA. Strains of *C. butyricum* are categorized based on their origin, encompassing human, animal, and other environmental sources. Research and applications have predominantly focused on strains originating from animals. Notably, only 10-20% of human intestines harbor *C. butyricum*, and the screening for strains of human origin continues to pose significant challenges (1)*.*

*C. butyricum* is widely acknowledged as a probiotic due to its myriad biological functions that enhance human health. For instance, it can promote cellular apoptosis by inhibiting the NF-κB pathway and modulate the structure and composition of the intestinal microbiota (2). The primary metabolic product of this bacterium, butyric acid, has been shown to regulate gut microbiota, reinforce intestinal barrier functions, mitigate inflammation, and stimulate cellular proliferation (3). Furthermore, certain strains of *C. butyricum* have demonstrated efficacy in regulating dysfunctions in lipid metabolism, including disturbances in glycolipid metabolism, impairment of liver and kidney functions, elevation of arterial blood pressure, and issues related to overweight, obesity, and insulin resistance (4).

The fundamental issue with obesity is an energy imbalance, which may result from excessive caloric intake, diminished energy expenditure, or both. Characterized by the excessive accumulation of adipose tissue, obesity engenders a rise in chronic low-grade inflammatory responses (5). As a multifactorial disease, obesity arises from a confluence of environmental and genetic factors. The prevalent consumption of high-fat diets and processed foods has disrupted the equilibrium of gut microbes, thereby escalating the risk of obesity. Additional contributing factors include inadequate exercise, societal pressures, and insufficient sleep, all of which have exacerbated the obesity epidemic and underscored the urgency of implementing multifaceted interventions to mitigate this health burden. Common obesity interventions include pharmacotherapy, surgical options, and dietary modifications (6). While these treatments may yield rapid results, they are often associated with side effects such as gastrointestinal disturbances and potential cumulative complications (7).

The equilibrium of the gut microbiome in humans is closely associated with overall health. A high-fat diet can alter the structure of the gut microbiome, notably by diminishing the prevalence of beneficial bacteria and augmenting that of deleterious bacteria, thus affecting the diversity of the gut microbial community. Such changes in the intestinal microbiota may contribute to the formation of fat deposits in the liver and the onset of related diseases, including fatty liver, inflammatory responses, and oxidative stress, which, in turn, may exacerbate obesity (8). Additionally, a high-fat diet can lead to a reduction in the population of butyrate-producing bacteria in the gut, thereby stimulating the production of pro-inflammatory factors and causing systemic inflammation, which could potentially lead to obesity (9).

*C. butyricum* is recognized for its role in maintaining gut health and ameliorating inflammatory bowel diseases. Recently, its application in combating obesity has started to emerge (10). In this study, a high-fat diet was employed to induce obesity in mice, followed by the oral administration of a live bacterial suspension of *C. butyricum* B-3 as an interventional treatment. This research examined physiological indicators, tissue sections, and gut microbiota to evaluate the anti-obesity and lipid-lowering properties of *C. butyricum* B-3. This report adheres to the guidelines provided in the Preferred Reporting Items for Microbiotherapy (PRIM) checklist (11) (Table 1).

**TABLE 1:** Evaluation of current FMT evidence in the anti-obesity effects of *Clostridium butyricum* B-3 based on PRIM 2024 checklist^*a*^

PRIM item	Status in studies
Indication	Preclinical: high-fat diet mice Clinical: route not defined
Diagnosis	Preclinical: 8 weeks of intervention to mice by high fat feed Clinical: route not defined
Disease condition	Severity: excessive weight gain noted in high-fat mice model; the kidney index and liver index of mice in the HFD group higher than those of ND group. Abnormal blood lipids and increase in intestinal inflammation levels in the HFD group Clinical comorbidities not specified
Delivery route	Animal: oral gavage with live bacteria Clinical trial: route not defined
Source	Animal donors: male C57BL/6J mice aged 8 weeks
Preparation for products	*C. butyricum* B-3 was isolated from healthy human gut and preserved in China General Microbiological Culture Collection Center
Classification	Treated as investigational therapy. The care and study of experimental mice followed the protocols of the Animal Care and Use Committee Classification for the products: not applicable
Dosage	Dose: the density of bacteria was adjusted by sterile saline to 5 × 10^6^ CFU/mL, 5 × 10^7^ CFU/mL, and 5 × 10^8^ CFU/mL, and the gavage dose was 200 µL Frequency: LCB: 10^6^ CFU/day; MCB: 10^7^ CFU/day; HCB: 10^8^ CFU/day
Formulation	Animal: during the intervention period, the body weight of live mice was measured weekly at the same time of the day. After 8 weeks of intervention, the mice were anesthetized, and blood was collected from the eyeballs. Then, the mice were immediately dislocated and executed Subsidiary material: normal feeds, high-fat feeds, anesthetics, etc.
Concomitant treatment	Animal preparation: normal group (12): standard feed; experimental group (13): high fat feed Treatments affecting the outcome: the low dose, medium dose, and high dose of *C. butyricum* B-3
Efficacy	Animal: *C. butyricum* B-3 exhibited good anti-obesity effects through a high-fat diet mice model Clinical: no efficacy data yet
Safety	Animal: no adverse events reported Clinical: no efficacy data yet

^*a*^ ND, normal diet; HFD, high-fat diet; LCB, low dose *C. butyricum* B-3; MCB, medium dose *C. butyricum* B-3; HCB, high dose *C. butyricum* B-3; PRIM, preferred reporting items for microbiotherapy.

## MATERIALS AND METHODS

### Chemicals

Normal and high-fat feeds were acquired from Beijing Keao Xieli Feed Co., Ltd. (Beijing, China). Solutions of 4% paraformaldehyde and hematoxylin-eosin for staining were sourced from Shanghai Yuanye Bio-Technology Co., Ltd. (Shanghai, China). Kits for testing triacylglycerols (TG), total cholesterol (TC), high-density lipoprotein cholesterol (HDL-C), and low-density lipoprotein cholesterol (LDL-C) were obtained from Nanjing Jiancheng Bioengineering Institute (Nanjing, China). All other chemicals, of the highest analytical grade, were procured from standard suppliers.

### Bacterial strains and culture conditions

*C. butyricum* B-3, isolated from a healthy human gut, was preserved at the China General Microbiological Culture Collection Center. The positive control strain, *C. butyricum* MIYAIRI 588, was isolated from MIY 588 live bacterial powder (Miyarisan Pharmaceutical Co., Ltd., Japan). Both strains were inoculated at a density of 6 × 10^7^ CFU/mL in reinforced clostridial medium (RCM) and cultured in an anaerobic incubator at 37°C.

### Growth curve and acid production capacity

Throughout the 24 h culture period, the pH and OD_600_ of the culture solutions were measured every 2 h to construct growth and acid production curves for the two strains.

### Determination of butyric acid production capacity

To assess the butyric acid production capacity, anaerobic fermentation was conducted at 37°C for 24 h using two bacterial strains. Post-fermentation, the broths were subjected to sonication for 30 min followed by centrifugation at 8,880 × *g* at 4°C for 20 min. The supernatant was then collected and filtered through a 0.22 µm membrane to prepare the samples. The concentration of butyric acid in these samples was quantified using high-performance gas chromatography, as described by He et al. (12).

### Determination of antioxidant capacity *in vitro*

#### Preparation of samples

The activated bacterial suspension was initially centrifuged at 2,656 × *g* for 10 min at 4°C to separate the supernatant and the bacterial pellet. Fermentation Supernatants (FS): The collected supernatant underwent a secondary centrifugation at 4,722 × *g* for 10 min at 4°C and was subsequently filtered under sterile conditions. Bacterial Cells (BC): The initially collected bacterial pellet was washed two to three times and resuspended in sterile physiological saline. The bacterial density was adjusted to 1.0 × 10^8^ CFU/mL.

#### Determination of the scavenging activity of DPPH radical

The scavenging activity of the DPPH radical was assessed following the methodology outlined by Yin et al. (14). To each 1 mL of FS and BC (prepared as described in “Preparation of samples,” above), 1 mL of a 0.2 mmol/L DPPH anhydrous ethanol solution was added. The mixtures were then incubated in a water bath at 37°C in darkness for 30 min. Following incubation, the mixtures were centrifuged at 2,656 × *g* for 10 min at 4°C, and the absorbance of the supernatant was measured at 517 nm. For zero adjustment, the sample solution was replaced with normal saline, and the DPPH anhydrous ethanol solution was replaced with anhydrous ethanol to serve as the blank. The DPPH radical scavenging rate was calculated using the following equation:


(1) $$A_{C}=\frac{A_{2}-A_{1}}{A_{2}}\times 100\%$$

Here, *A*_C_ represents the DPPH radical scavenging rate, *A*_1_ is the absorbance of the reaction system with the sample solution, and *A*_2_ is the absorbance of the reaction system where normal saline replaces the sample solution.

#### Determination of hydroxyl radical scavenging activity

The hydroxyl radical (OH) scavenging activity was determined by a slightly modified method of Lombardo et al. (15). Initially, 1,10-phenanthroline (1 mL, 2.5 mmol/L) and sterile phosphate-buffered saline (PBS; 1 mL, 0.1 mol/L, pH 7.2) were added to 1 mL each of FS and BC as prepared in “Preparation of samples,” above. Subsequently, FeSO_4_ (1 mL, 2.5 mmol/L) and H_2_O_2_ (1 mL, 20 mmol/L) were introduced to the mixture. The reaction was conducted in a water bath maintained at 37°C for 90 min. The absorbance of the resultant mixture was measured at 536 nm. The capacity for hydroxyl radical scavenging was quantified according to the following equation:


(2) $$A_{C}=\frac{A_{1}-A_{2}}{A_{3}-A_{2}}\times 100\%$$

where *A*_C_ represents the hydroxyl radical scavenging rate, *A*_1_ is the absorbance of the sample solution reaction system, *A*_2_ is the absorbance of the control reaction system using physiological saline instead of the sample solution, and *A*_3_ is the absorbance of the control reaction system using distilled water instead of hydrogen peroxide.

#### Determination of the reducing ability

The reducing ability of the samples was determined following the procedure outlined by Dai et al. (16) with slight modifications. Sample FS and BC, obtained as described in “Preparation of samples,” above, were each mixed with an equal volume of potassium ferricyanide (1%) and PBS buffer (0.2 mol/L, pH 6.6). The reaction was halted by the addition of trichloroacetic acid (10%, 0.5 mL) after incubating in a water bath at 50°C for 20 min. Following centrifugation at 664 × *g* for 10 min at 4°C, 1 mL of the supernatant from each sample was combined with 1 mL of ferric chloride solution (0.1%). The mixture was then allowed to stand for 10 min at 37°C before measuring the absorbance at 710 nm. The reducing ability was quantified using the following formula (3):


(3) $$Reducing\;ability=\frac{A_{1}-A_{0}}{A_{1}}\times 100\%$$

where *A*_1_ is the absorbance of the sample solution reaction system, *A*_2_ is the absorbance of the control reaction system using physiological saline instead of the sample solution, and *A*_0_ is the baseline absorbance of the reaction system with physiological saline alone.

### Lipid-lowering capacity *in vitro*

#### Bile-acid salt binding capacity

Sodium taurocholate and sodium glycocholate standard solutions (0.012, 0.06, 0.12, 0.18, 0.24, and 0.3 mmol/L) were freshly prepared using a phosphate buffer (0.1 mol/L, pH 6.3). Each 2.5 mL of the standard solution was combined with 7.5 mL of H₂SO₄ (60%) and rapidly cooled following a 20 min incubation in a water bath maintained at 70°C. Subsequently, the absorbance was measured at 387 nm, and a standard curve was constructed by plotting concentration on the horizontal axis against absorbance on the vertical axis.

The bile-acid salt binding capacity was assessed using a modified method originally described by Kahlon et al. (17). This involved the use of FS and BC, prepared as outlined in “Preparation of samples,” above. A mixture of 3 mL FS/BC, 3 mL pepsin (10 g/L), and 1 mL hydrochloric acid solution (0.01 mol/L) was incubated with continuous temperature-controlled shaking for 1 h at 37°C. The pH of the mixture was adjusted to 6.3 using a sodium hydroxide solution (0.1 mol/L), followed by the addition of 4 mL trypsin (10 g/L). This mixture was then incubated under similar conditions for an additional hour. Afterward, 4 mL of either sodium taurocholate (0.5 mmol/L) or sodium glycocholate (0.4 mmol/L) was added, and the incubation continued at 37°C for another hour. The mixtures were then centrifuged (1180 × *g*, 20 min, 4°C), and the supernatants were collected for analysis using a colorimetric method involving sulfuric acid. The bile-acid salt binding capacity was quantified according to Equations (4) and (5) and 5:


(4) $$Sodium\;glycocholate\;\left(mmol/mL\right)=\frac{N_{1}-N_{2}}{V}\times A$$

(5) $$Sodium\;taurocholate\;\left(mmol/mL\right)=\frac{N_{3}-N_{4}}{V}\times A$$

where *N*_1_ represents the initial amount of sodium glycocholate added (mmol), *N*_2_ is the amount of sodium glycocholate remaining (mmol), *N*_3_ is the initial amount of sodium taurocholate added, and *N*_4_ is the amount of sodium taurocholate remaining. The volume of the sample added is denoted by *V* (mL), and *A* represents sample dilutions.

#### Inhibition of pancreatic lipase

The inhibition of pancreatic lipase assessed using a method adapted from Kim et al. (18). Initially, 4 mL of a polyvinyl alcohol glyceryl trioleate emulsion was combined with 5 mL of PBS buffer (0.025 mol/L, pH 7.4) and incubated in a water bath at 40°C for 10 min. Subsequently, 1 mL of a tryptic lipase solution (2 mg/mL) was added, and the mixture was incubated at the same temperature for an additional 15 min. The reaction was halted by introducing 15 mL of 95% ethanol. For the control, 1 mL of pancreatic lipase solution was added at the end of the procedure, with all other steps replicating the experimental protocol. The mixture was then titrated using phenolphthalein indicator and 0.05 mol/L sodium hydroxide.

For a parallel set of measurements, 4 mL of emulsion was treated with 5 mL of PBS buffer in a 40°C water bath for 10 min. Here, 1 mL of FS/BC was introduced to the system and incubated under similar conditions for 10 min. The subsequent steps were consistent with those described previously. The activity of pancreatic lipase was quantified using the following formula:


(6) $$U\left(IU\right)=\frac{1000\times \left(V_{1}-V_{2}\right)\times C}{WT}$$

where *U* represents the enzyme activity per gram of pancreatic lipase in International Units (IU), *V*_1_ is the volume of NaOH consumed by the sample group I in mL, *V*_2_ is the volume consumed in the control/blank group, *C* is the concentration of the NaOH standard solution in mol/L, *W* denotes the quantity of pancreatic lipase added in grams, and *T* is the duration of the reaction following the addition of pancreatic lipase in minutes.

Inhibition of pancreatic lipase was calculated using the following:


(7) $$Inhibition\;of\;pancreatic\;lipase\left(\%\right)=1-\frac{Enzyme\;activity\;after\;inhibition}{\;Enzymatic\;activity\;before\;inhibition}$$

#### Cholesterol-reducing rate

The cholesterol-reducing rate was determined following a modified protocol from Luo et al. (19). The bacterial strain was cultured in RCM cholesterol medium at a 2% concentration and incubated at 37°C for 12 h. A blank control, consisting of medium without any inoculated bacteria, was also maintained under the same conditions. Post incubation, the supernatant was collected for analysis after centrifugation at 1,180 × *g* and 4°C for 10 min. The cholesterol content in the fermentation supernatant was measured using a TC assay kit. The cholesterol-reducing rate was then calculated according to the following:


(8) $$Cholesterol\;reducing\;rate\;\left(\%\right)=1-\frac{A}{B}\times 100\%$$

where *A* is the cholesterol content in the sample group measured in μmol/L, and *B* is that in the control group.

### Animal experiment design

**Fig 1:**
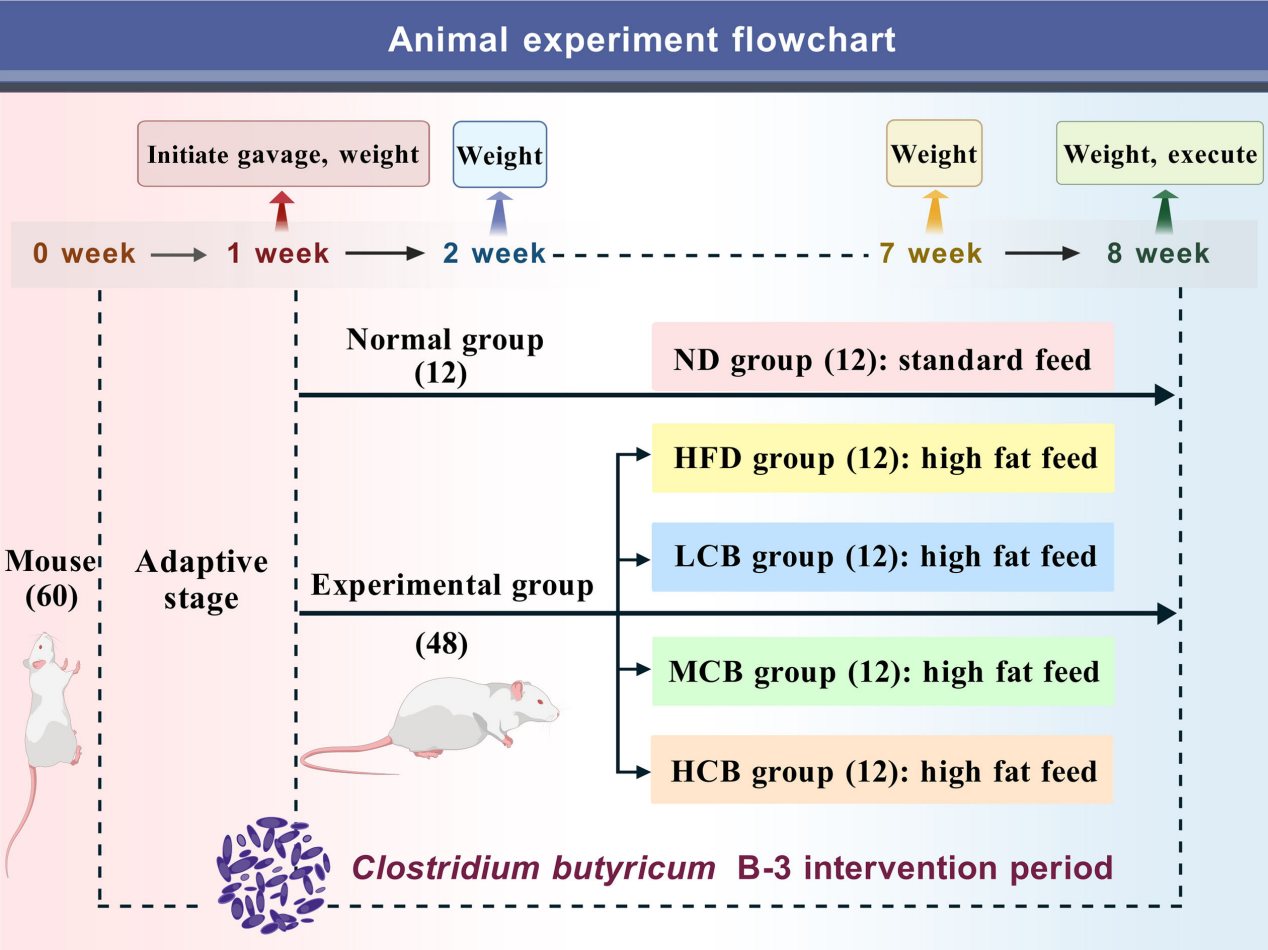
Animal experiment flowchart.

Male C57BL/6J mice, aged 8 weeks, were procured from the Henan Animal Experiment Center. The Laboratory Animal Centre was licensed under the number SCXK (Yu) 2022-0001. All mice were maintained in an environment with controlled temperature and humidity (24 ± 2°C, 50% ± 10%) and subjected to a 12 h light/dark cycle. The experimental design is depicted in Fig. 1. Following a 1-week acclimatization period, the mice were randomly allocated into five groups, each consisting of 12 mice housed four per cage. The intervention involved administering live bacteria via oral gavage for a duration of 8 weeks. The bacterial density was standardized using sterile saline to concentrations of 5 × 10^6^ CFU/mL, 5 × 10^7^ CFU/mL, and 5 × 10^8^ CFU/mL, with each gavage dose being 200 µL. Thus, the final intervention doses were 10^6^ CFU/day (low dose), 10^7^ CFU/day (medium dose), and 10^8^ CFU/day (high dose). The groups and intervention methods are presented in Table 2. Throughout the 8 week intervention period, the body weight of the mice was recorded weekly at the same time each day (9:00 AM). The weight gain was calculated using the formula provided in Equation (9).

**TABLE 2:** Experimental animal groups

Group	Gavage solution/dose	Fodder
Normal diet (ND）	Sterile saline /200 µL/day	Ordinary fodder
High-fat diet (HFD)	Sterile saline /200 µL/day	High-lipid fodder
Low dose *C. butyricum* B-3 (LCB）	Bacterial suspension/10^6^ CFU/day	High-lipid fodder
Medium dose *C. butyricum* B-3 (MCB）	Bacterial suspension/10^7^ CFU/day	High-lipid fodder
High dose *C. butyricum* B-3 (HCB）	Bacterial suspension/10^8^ CFU/day	High-lipid fodder


(9) weight gain (g) =weight (week 8)− weight (week 0)

### Sample collection

Following the 8 week intervention, the mice were anesthetized, and blood was drawn from the orbital sinus. Subsequently, cervical dislocation was promptly performed to euthanize the animals. The collected blood samples were centrifuged (2,000 × *g*, 15 min, 4°C) and the serum was preserved at −20°C. The mice were then dissected, and their kidneys, liver, and spleen were rinsed with saline (0.9%), drained, and weighed. The livers were fixed in 4% paraformaldehyde for subsequent histopathological examination. Colonic feces were collected on a sterile super-clean bench, immediately transferred to sterile centrifuge tubes, and stored at −80°C. The visceral index was calculated as described in Equation (10).


(10) $$Visceral\;index\;(\%)=\frac{Weight\;of\;organ\;(g)}{Weight\;of\;mice\;(kg)}\times 100\%$$

Liver histopathology sections were prepared in accordance with the protocol outlined by Xu et al. (20). The liver tissues were fixed in 4% paraformaldehyde, embedded in paraffin, sectioned (4–6 µm thickness), and stained with hematoxylin-eosin (HE). The morphology of the liver was then examined microscopically.

### Measurement of lipid metabolism indicators and inflammatory factors

The levels of triglycerides (TG), total cholesterol (TC), high-density lipoprotein cholesterol (HDL-C), low-density lipoprotein cholesterol (LDL-C), interleukin-6 (IL-6), interleukin-10 (IL-10), and tumor necrosis factor-alpha (TNF-alpha) were determined following the protocols specified in the respective assay kits.

### Analysis of intestinal flora in mice

Total genomic DNA was extracted from the feces of mouse colons using the CTAB/SDS method. The extracted DNA was subsequently amplified via PCR and purified with a gel extraction kit. Sequencing libraries were prepared using the TruSeq DNA PCR-Free Sample Preparation kit (Illumina, USA). These libraries were assessed using the Qubit 2.0 Fluorometer (Thermo Scientific) and the Agilent Bioanalyzer 2100 systems, followed by paired-end sequencing, splicing, and quality control based on the Illumina NovaSeq sequencing platform. This process yielded Effective Tags suitable for further analysis. OTUs were clustered at 97% similarity, and species annotation of OTU sequences was performed using the mothur method to determine sample composition. Visualization of species composition was achieved with QIIME2.

### Determination of short-chain fatty acids in mouse feces

The quantification of short-chain fatty acids (SCFAs) in mouse feces was conducted via high-performance gas chromatography, as described by He et al. (12). The analysis utilized an Agilent 7890B GC system equipped with an Agilent J&W GC Column DB-FFAP122-3232 (30 m × 0.25 mm × 0.25 µm). Nitrogen served as the carrier gas, and a hydrogen flame ionization detector was employed at a temperature of 300°C. Samples were introduced in an automated injection mode with an injection temperature of 300℃, a split ratio of 15:1, and a sample volume of 1 µL.

### Statistical analysis

All experiments were conducted in at least triplicate, with results presented as means ± standard deviation. Data visualization and analysis were performed using Origin 2018 64-bit software (OriginLab Co., Northampton, MA, USA). Statistical significance was assessed using one-way ANOVA at a significance level of *P* < 0.05, conducted with SPSS 25 Statistics (SPSS, Inc., Chicago, IL, USA).

Animal experiments included at least six parallel measurements per sample, with results also presented as means ± standard deviation. To examine the significance of community structure differences among groups, the adonis and anosim functions in QIIME2 were utilized. Identification of significantly different species at each taxonomic level (Phylum, Genus) was performed using MetaStat and *t*-test analyses in R software (Version 3.5.3). Furthermore, LEfSe analysis (LDA score threshold: 4) was conducted using LefSe software (Version 1.0) to identify potential biomarkers.

## RESULTS AND DISCUSSION

### Proliferation ability, acid production ability, and butyric acid production ability

**Fig 2:**
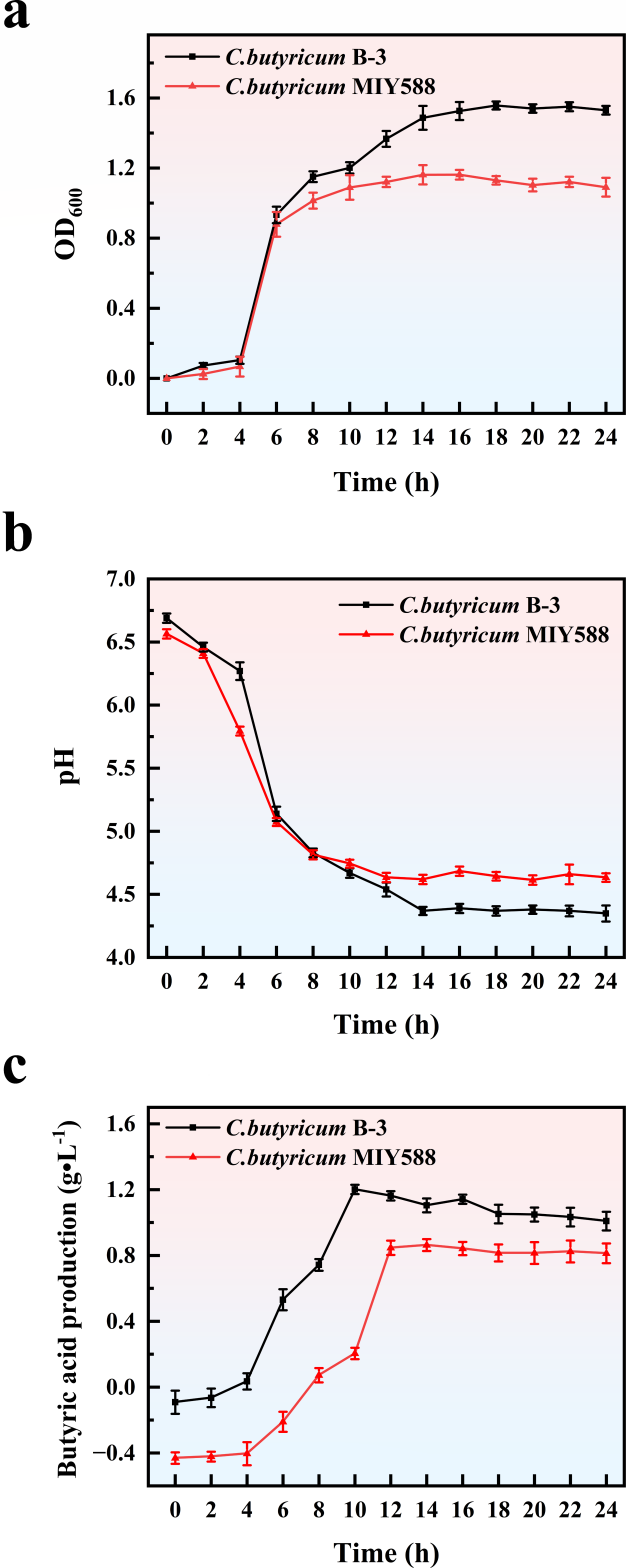
Growth curve, acid production ability, and butyric acid production (**a**, growth curve; **b**, acid production curve; **c**, butyric acid production).

The growth and acid production capacity of probiotics are critical indicators for assessing their probiotic properties. The growth curves of the two strains are depicted in Fig. 2a. It is apparent that under identical cultivation conditions, the growth patterns of *C. butyricum* B-3 and *C. butyricum* MIY588 were consistent. Specifically, the initial 4 h served as the growth acclimation period, followed by a rapid growth phase from 4 to 10 h. Subsequently, the cultures entered a stable phase characterized by a slow increase in bacterial biomass. Notably, after 6 h, *C. butyricum* B-3 demonstrated significantly enhanced growth vitality compared to *C. butyricum* MIY588.

*C. butyricum* is known to produce various short-chain fatty acids during its growth, leading to a reduction in the pH of the growth environment (21). As illustrated in Fig. 2b, the pH curves of the fermentation broth for both strains correlated with the trends observed in their growth curves. After 14 h, the pH curves reached a stable phase. The pH value of *C. butyricum* B-3 stabilized at approximately 4.3, whereas that of *C. butyricum* MIY588 stabilized around 4.6, indicating that *C. butyricum* B-3 produced more acid than *C. butyricum* MIY588.

*C. butyricum* is renowned for its high production levels of butyric acid, principally through the enzymatic activities of butyrate kinase and butyryl-CoA (22). Butyric acid possesses numerous functional properties, predominantly related to energy metabolism and cell signaling (23). In terms of energy metabolism, butyric acid participates in cellular energy production by contributing to the Krebs cycle and is also converted into acetyl-CoA in the liver, which is further involved in the beta-oxidation of fatty acids (24). In cell signaling, butyric acid helps maintain intracellular acid-base balance, stimulates the production of epidermal growth factor, and enhances anti-inflammatory factors, which, in turn, boosts antioxidant and anti-inflammatory capacities, thereby protecting cells from oxidative stress and inflammatory damage (24). Consequently, butyric acid production is a primary indicator for assessing the probiotic efficacy of *C. butyricum*.

As demonstrated in Fig. 2c, during the fermentation process, both strains showed a rapid rate of butyric acid production between 4 and 12 h. Subsequently, the butyric acid production of *C. butyricum* B-3 eventually stabilized at around 1.0 g/L and that of *C. butyricum* MIY588 stabilized at approximately 0.8 g/L.

In conclusion, *C. butyricum* B-3 exhibited superior growth activity, acid production, and butyric acid production compared to *C. butyricum* MIY588, underscoring its potential as a probiotic strain.

### Antioxidant activity and lipid-lowering capacity of *C. butyricum* B-3 *in vitro*

#### Antioxidant activity of *C. butyricum* B-3

**Fig 3:**
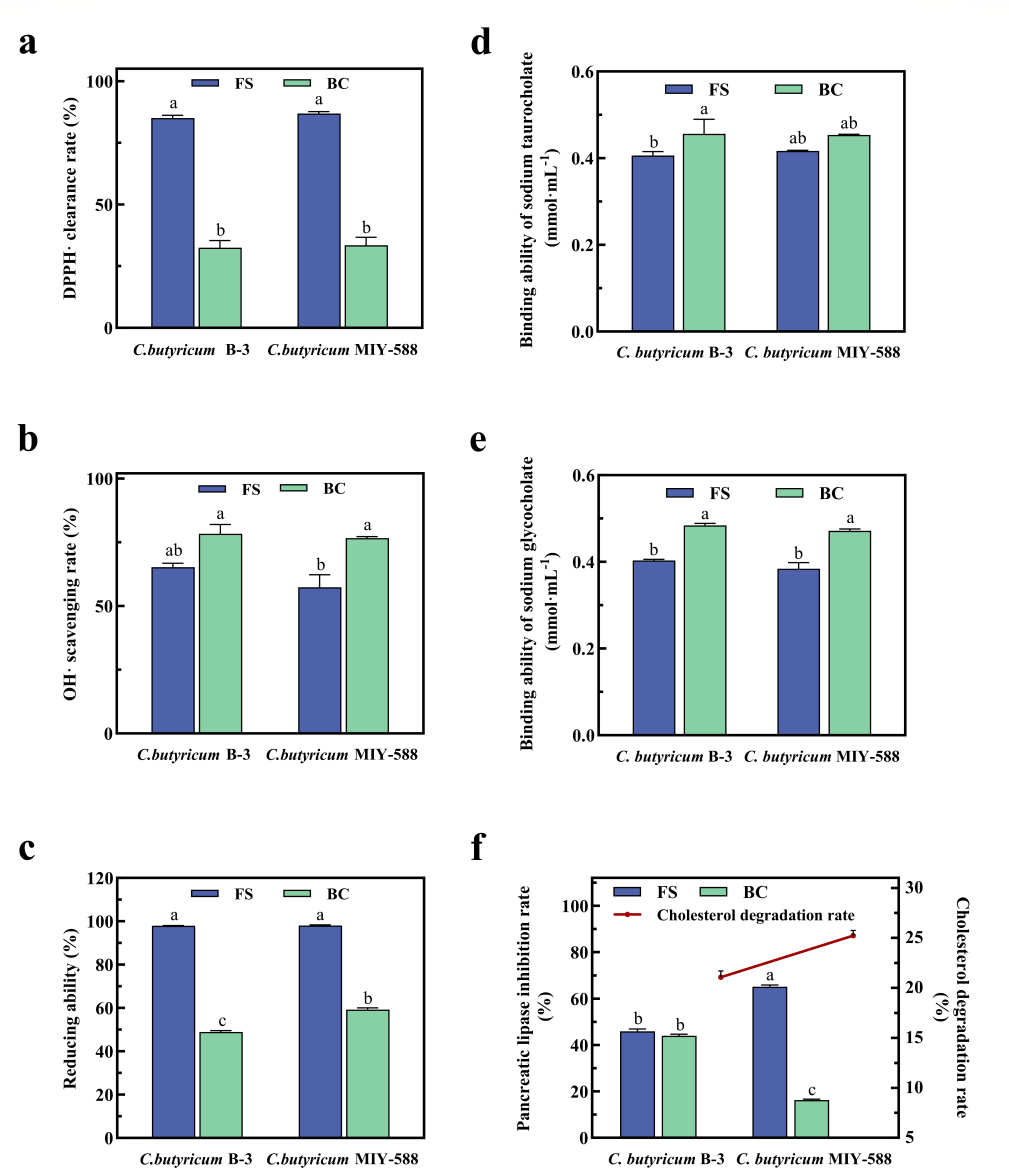
The antioxidant activity of strains and *in vitro* lipid-lowering capacity of strains (**a**, DPPH· clearance rate; **b**, OH· scavenging rate; **c**, reducing ability; **d**, binding ability of sodium taurocholate; **e**, binding ability of sodium glycocholate; **f**, pancreatic lipase inhibition rate) (FS, fermentation supernatant; BC, bacterial cells).

Antioxidants are capable of reducing the stable 2,2-diphenyl-1-picrylhydrazyl (DPPH) free radicals into 2,2-diphenyl-1-picrylhydrazine (DPPH-H), resulting in a measurable color reaction. The intensity of the color produced is inversely proportional to the antioxidant content (25). As illustrated in Fig. 3a, the capacity of *C. butyricum* B-3 and *C. butyricum* MIY588 to scavenge DPPH radicals was comparable, suggesting that both strains captured a similar number of DPPH single electrons. Furthermore, FS demonstrated a superior ability to scavenge DPPH radicals, significantly outperforming BC (*P* < 0.05). This enhanced performance may be attributed to metabolites secreted by *C. butyricum* during fermentation, such as butyric acid and hydrogen gas found in the fermentation supernatant, which are known to mitigate oxidative stress and scavenge free radicals (26).

Free radical-induced tissue damage plays a role in the pathogenesis of various diseases. The antioxidant properties of *C. butyricum* can impede the diffusion of free radicals, bolster immune capability, and augment the protective response of normal cells against tissue damage (27). As depicted in Fig. 3b, a comparison of the hydroxyl radical scavenging rates between FS and BC from strains *C. butyricum* B-3 and *C. butyricum* MIY588 indicated that BC was more effective than FS. This suggests that the primary mechanism for hydroxyl radical clearance by these strains is through the bacterial suspension, likely involving interaction with free radicals on the bacterial surface or within the bacteria themselves. This activity is facilitated by live bacterial cells and is linked to both the fermentation broth and the metabolites produced during the fermentation process. Han et al. (28) observed that *C. butyricum* could inhibit hydroxyl radicals and moderately decrease hydrogen peroxide concentrations, a capability likely associated with the hydrogen gas produced as a metabolic byproduct of *C. butyricum*.

As depicted in Fig. 3c, *C. butyricum* MIY588 demonstrated a greater reductant capacity than *C. butyricum* B-3. Among these strains, the reductant ability of FS was significantly stronger (*P* < 0.05) than that of BC, suggesting that the principal active substances contributing to this capacity are predominantly found in FS. Wang et al. (29) corroborated this finding by analyzing the reductant capacities of both the fermentation broth and the bacterial suspension of lactic acid bacteria. Additionally, Mun et al. (30) reported that the fermentation supernatant of *C. butyricum* contains copious antibacterial and anti-inflammatory agents, including short-chain fatty acids and other metabolites. In the current study, three indicators—DPPH radical scavenging rate, OH radical scavenging rate, and reducing ability—were employed to assess the antioxidant potential of *C. butyricum* B-3 and *C. butyricum* MIY588. The results demonstrated that the antioxidant capacities of *C. butyricum* B-3 were comparable to those of *C. butyricum* MIY588, indicating promising prospects for the application of *C. butyricum* B-3. However, further research is necessary to elucidate the precise antioxidant mechanisms of *C. butyricum* B-3.

#### Lipid-lowering capacity of C. butyricum B-3

*Lactobacillus* species have the ability to bind to bile-acid salts, thereby interrupting the “liver-intestinal cycle.” As a result, the liver is stimulated to convert cholesterol into bile-acid salts, thereby reducing cholesterol levels and exerting a lipid-lowering effect (31). In this study, sodium glycocholate and sodium taurocholate were chosen as experimental agents due to their differing structures and hydrophilicity, which accurately reflect the binding capacity of the active components of lactic acid bacteria to bile-acid salts (32). As illustrated in Fig. 3d and e, the binding capacities of BC from *C. butyricum* B-3 and *C. butyricum* MIY588 with sodium glycocholate and sodium taurocholate were superior to those of FS, indicating that the lipid-lowering mechanism of *C. butyricum* predominantly involves the direct interaction between the bacteria and bile-acid salts. This interaction likely enhances the inhibition of lipid emulsification and absorption, thus effectively reducing blood lipid levels. In summary, the lipid-lowering effects of the two strains were comparable, suggesting that *C. butyricum* B-3 also holds significant potential for development into lipid-lowering products.

Pancreatic lipase, an enzyme integral to the hydrolysis of dietary fats in humans, plays a pivotal role in this metabolic process. This enzyme facilitates the breakdown of dietary fats into free fatty acids, glycerol, and mono- or di-glycerol esters. By inhibiting pancreatic lipase activity, one can effectively reduce the hydrolysis of dietary triglycerides, thereby decreasing lipid absorption and contributing to the reduction of blood lipid levels (33). As illustrated in Fig. 3f, the BC of *C. butyricum* B-3 exhibited a significant inhibitory effect on pancreatic lipase, with a 44.02% inhibition rate that was statistically higher (*P* < 0.05) than that of the positive control. Furthermore, the FS of *C. butyricum* MIY588 displayed the most potent inhibition of pancreatic lipase. Comprehensive analysis of FS and BC data revealed that *C. butyricum* B-3 was more effective in inhibiting pancreatic lipase.

Elevated serum cholesterol is a primary indicator of hyperlipidemia, obesity, and various cardiovascular and cerebrovascular diseases (34). Previous research has demonstrated that probiotics, such as *lactobacilli*, possess cholesterol-lowering properties although the efficacy of *C. butyricum* in this regard remains under investigation (35). Figure 3f shows that the cholesterol reduction by *C. butyricum* MIY588 was 25.22%, surpassing the 21.07% reduction by *C. butyricum* B-3. Meng et al. (34) observed that *L. plantarum* BHP03 and LGG reduced cholesterol by 20%–35% *in vitro*, a range comparable to the reductions achieved by the *C. butyricum* strains in this study, suggesting that *C. butyricum* may, indeed possess cholesterol-lowering capabilities.

### Effects of *C. butyricum* B-3 on the body weight and organ health in mice on a high-fat diet

**Fig 4:**
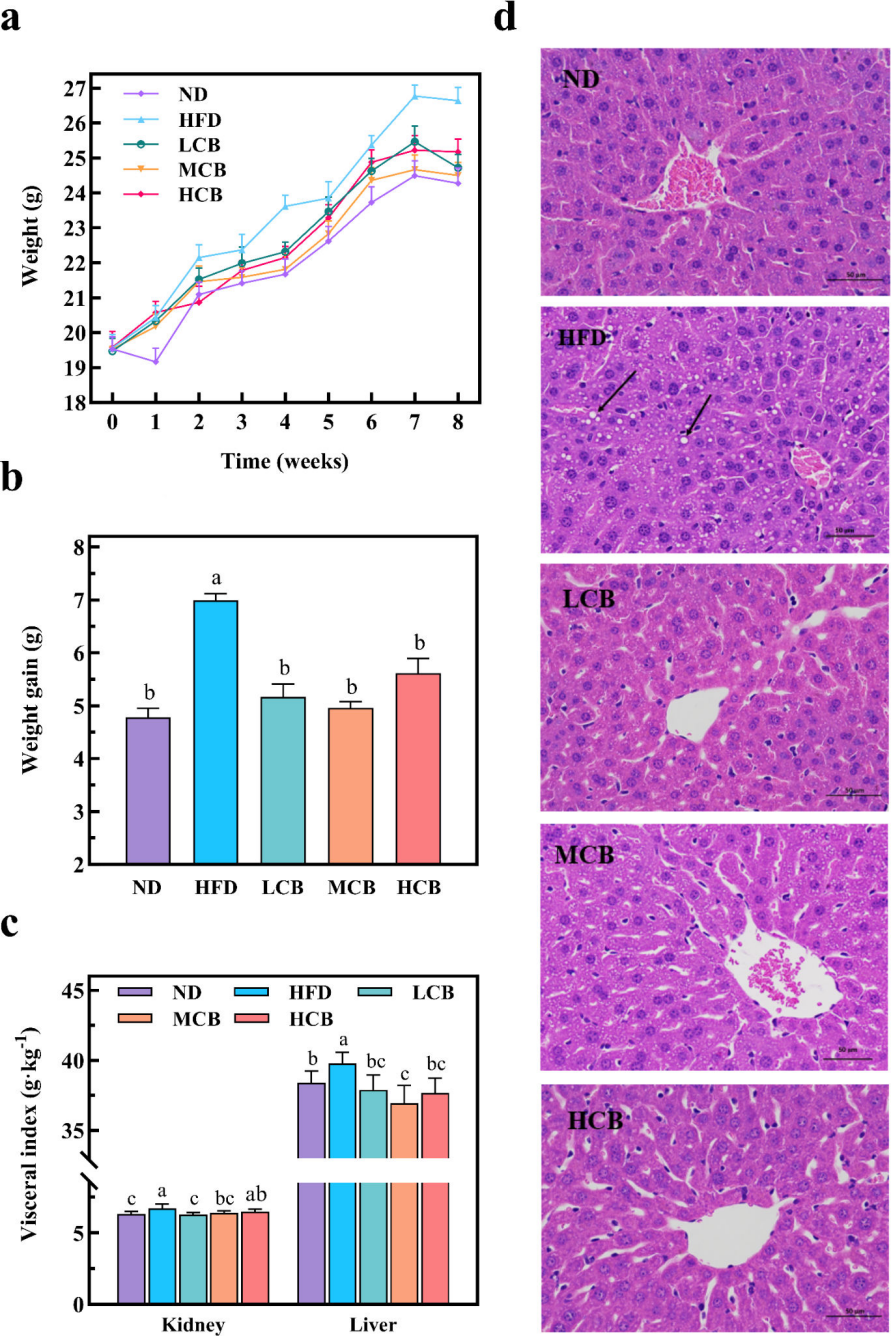
Effects of *C. butyricum* B-3 on the body weight (**a**), weight gain (**b**), organ index (**c**), and liver tissue lesions (400×) (**d**) of high-fat diet mice. The arrows expressed the differences (large intracytoplasmic vacuoles formed by the fusion of a large number of small lipid droplet vacuoles) (ND, normal diet; HFD, high-fat diet; LCB, low dose *C. butyricum* B-3; MCB, medium dose *C. butyricum* B-3; HCB, high dose *C. butyricum* B-3).

Body weight serves as the most direct measure of obesity in mice. The body weight changes in mice subjected to *C. butyricum* B-3 intervention were depicted in Fig. 4a and b. Throughout the study, all groups exhibited an upward trend in body weight. Notably, the HFD group experienced a significantly more rapid increase in body weight compared to the other four groups (Fig. 4a). After the 8-week intervention, the weight gain in the HFD group was significantly greater than in the other groups, with no notable differences among the latter (Fig. 4b). These findings underscore that a high-fat diet, indeed prompts excessive weight gain, whereas intervention with *C. butyricum* B-3 can mitigate the weight gain associated with such a diet.

A prolonged high-fat diet can lead to the deposition of lipids in various organs, thereby impairing organ functionality, inducing tissue inflammation, and causing organ hypertrophy (36). Organ indices are commonly utilized to quantify the extent of organ damage. As depicted in Fig. 4c, both the kidney and liver indices in mice subjected to a HFD were significantly elevated compared to those in the ND group (*P* < 0.05). This phenomenon may be attributable to the high cholesterol content in such diets, as Ma et al. (37) observed that excessive cholesterol, when not fully metabolized, accumulates in tissues and organs, leading to their enlargement. Conversely, in groups receiving low, medium, and high concentrations of *C. butyricum* B-3 (LCB, MCB, and HCB, respectively), both kidney and liver indices were significantly reduced (*P* < 0.05). These findings suggest that supplementation with *C. butyricum* B-3 mitigates the organ enlargement induced by a high-fat diet.

Obesity is frequently associated with disrupted lipid metabolism in the liver, resulting in lipid accumulation and the manifestation of fatty liver, characterized by fatty alterations in hepatic cells. Examination of liver tissue sections (Fig. 4d) revealed that the liver architecture in the ND group was well-organized, with distinct boundaries between hepatocyte bundles. In contrast, liver cells in the HFD group displayed numerous prominent white vacuoles, a disorganized cellular arrangement, and enlarged intercellular spaces. The cytoplasm exhibited a pronounced granular texture due to the accumulation of lipid droplets (38). Notably, the LCB, MCB, and HCB groups showed a marked reduction in both the size and number of these fat droplets. Similar effects on fatty liver mitigation by high-fat diets have been reported with other strains, such as *C. butyricum* B1 and *C. butyricum* MIYAIRI 588 (39, 40).

This study confirms the efficacy of *C. butyricum* B-3 in reducing excessive weight gain and organ lipid accumulation attributable to a high-fat diet.

### Effects of *C. butyricum* B-3 on lipid metabolism and inflammatory factors in mice fed a high-fat diet

The concentration of lipids in the bloodstream is a critical indicator for assessing metabolic syndrome in humans. Elevated blood lipid levels, commonly associated with a high-fat diet, reflect disruptions in lipid metabolism and are typically marked by increased levels of total cholesterol (TC), total triglycerides (TG), and low-density lipoprotein (LDL-C), alongside reduced high-density lipoprotein (HDL-C) levels (41).

**Fig 5:**
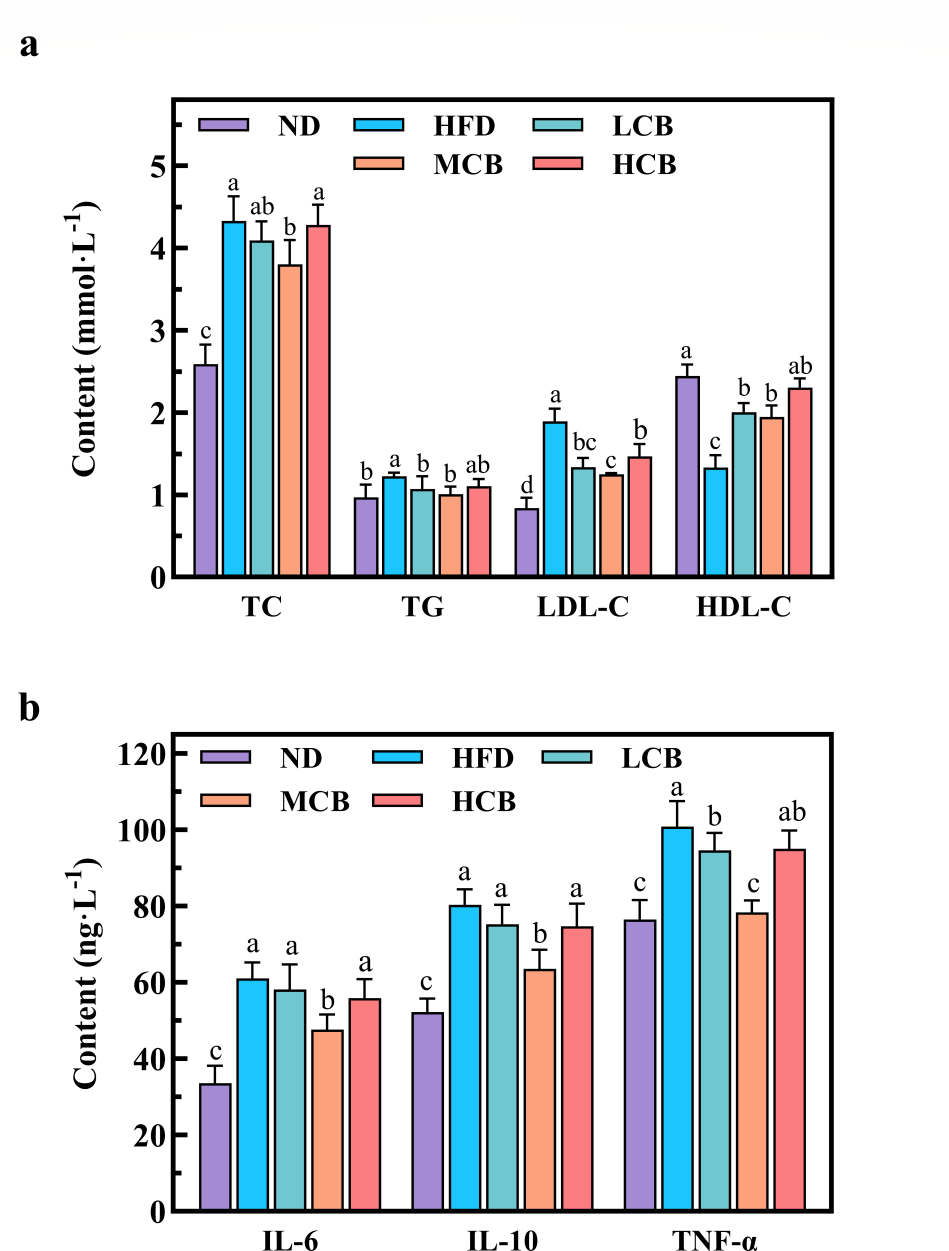
Effects of *C. butyricum* B-3 on the blood lipid related indexes (**a**) and the serum inflammatory cytokine levels (**b**) of high-fat diet mice (ND, normal diet; HFD, high-fat diet; LCB: low dose *C. butyricum* B-3; MCB, medium dose *C. butyricum* B-3; HCB, high dose *C. butyricum* B-3).

As illustrated in Fig. 5a, serum TC, TG, and LDL-C concentrations were significantly elevated in the HFD group compared to the ND group (*P* < 0.05). This elevation signifies that a high-fat diet not only disrupts normal lipid metabolism but also induces metabolic disorders in mice (42). Conversely, in the mice treated with *C. butyricum* B-3 (MCB groups), there was a significant reduction in the levels of TC, TG, and LDL-C (*P* < 0.05). These findings imply that *C. butyricum* B-3 intervention ameliorates lipid metabolic disorders induced by a high-fat diet, potentially through the mechanisms discussed in the Lipid-lowering capacity of *C. butyricum* B-3 section concerning its *in vitro* lipid-lowering capabilities.

HDL-C primarily facilitates the transport of cholesterol from various tissues back to the liver, where it is metabolized. This process is crucial for lowering blood cholesterol levels and reducing the incidence of atherosclerosis, hence HDL-C is often referred to “good cholesterol” (43). In the HFD group (Fig. 5a), HDL-C levels were markedly lower compared to the ND group (*P* < 0.05). Intervention with *C. butyricum* B-3 substantially increased HDL-C levels (*P* < 0.05), with values in the HCB group nearing those observed in the ND group. These outcomes demonstrate that *C. butyricum* B-3 effectively mitigates dyslipidemia in mice fed a high-fat diet.

Furthermore, a high-fat diet is known to elevate intestinal inflammation levels, which can adversely affect overall health and contribute to weight gain (44). Cytokines play essential roles in the pathophysiology of inflammation, with specific cytokines such as interleukin-2 (IL-2), interleukin-6 (IL-6), interleukin-9 (IL-9), interleukin-10 (IL-10), and tumor necrosis factor-α (TNF-α) being particularly significant (45). Notably, IL-6 and TNF-α are known to exacerbate inflammatory responses, whereas IL-10 serves an anti-inflammatory function within the organism (46).

As illustrated in Fig. 5b, the HFD group exhibited significantly elevated levels of IL-6, IL-10, and TNF-α compared to the ND group (*P* < 0.05). Conversely, the levels of inflammatory markers in the MCB group were consistently lower than those observed in the HFD group. A medium dose of *C. butyricum* B-3 (10^7^ CFU/day) was found to ameliorate inflammation induced by a high-fat diet. Hayashi et al. (47) reported that *C. butyricum* could mitigate the severity of colonic inflammation by suppressing the excessive secretion of IL-10 and facilitating the repair of damaged intestinal tissue.

### Effects of *C. butyricum* B-3 on the intestinal flora and short-chain fatty acids content in high-fat diet mice

#### OTU analysis and UPGMA clustering tree

**Fig 6:**
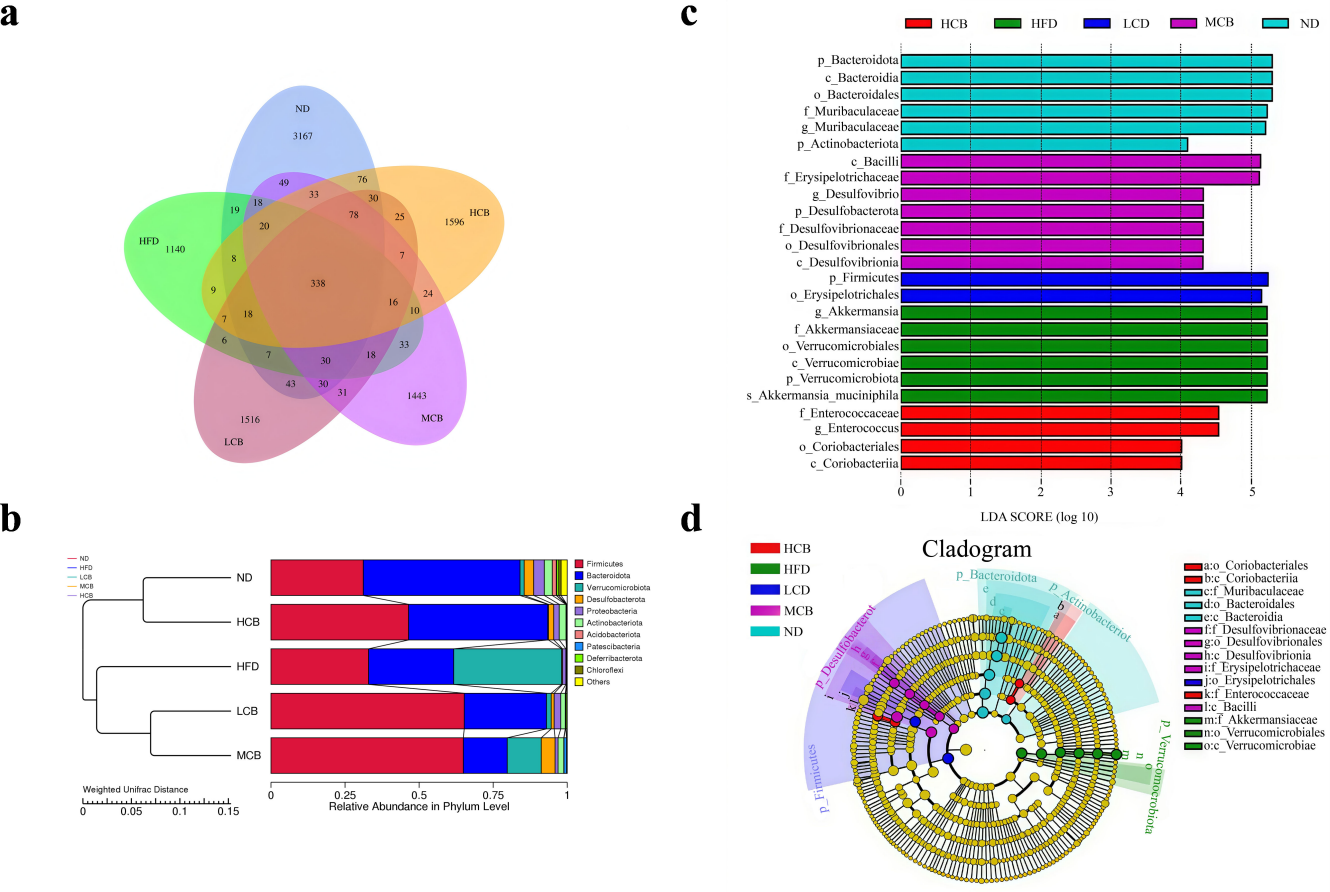
OTU analysis (**a**), UPGMA clustering tree (**b**), and LEfSe multilevel species difference analysis. (**c**, LDA value distribution bar chart; **d**, evolutionary branch chart) (ND, normal diet; HFD, high-fat diet; LCB, low dose *C. butyricum* B-3; MCB, medium dose *C. butyricum* B-3; HCB, high dose *C. butyricum* B-3).

Prior research has established that high-fat diets adversely impact the abundance and diversity of intestinal flora in mice (34). Analysis via a Venn diagram revealed the number of OTUs that were shared and unique across multiple samples (Fig. 6a). A total of 338 OTUs were common across the 5 groups, indicating the existence of a core microbiota in the murine gut. Notably, the HFD group possessed the fewest OTUs (1,140), which underscores the detrimental effect of a high-fat diet on microbial diversity. However, intervention with *C. Butyricum* B-3, particularly at a high dosage (10^8^ CFU/day), significantly enhanced species diversity.

To assess the similarity of intestinal flora across various groups, a UPGMA clustering tree was constructed to illustrate the composition and relative abundance of species within the samples. As depicted in Fig. 6b, the ND group and the HCB group formed a cluster, indicating a highly similar species composition structure between these groups. However, the medium and low dose interventions grouped with the HFD group, suggesting that lower dosages were less effective at influencing the gut microbiota of mice.

#### LEfSe multilevel species difference analysis

LEfSe (linear discriminant analysis effect size, LDA effect size) enables comparisons between multiple groups and subgroup analyses within groups to identify species with significant differences in abundance. The LEfSe analysis incorporates an LDA value distribution bar chart (Fig. 6c) and an evolutionary branch chart (Fig. 6d). The LDA value distribution bar chart illustrates biomarkers that show statistical differences between groups, with the length of each bar indicating the magnitude of the impact of different species (48).

In the ND group, the differential species were primarily from the phylum *Bacteroideta*, *Actinobacteria,* and the *Muribaculaceae* family. In contrast, the HFD group exhibited differential species such as *Verrucomicrobiota* and *Akkermansiaceae*. The LCB group was characterized by an abundance of *Firmicutes* and their subordinate *Erysipelotrichales*, whereas the MCB group included *Bacilli*, *Erysipelotrichaceae*, and *Desulfobacterota*. The HCB group’s differential species were *Enterococcus* and *Coriobacteria*.

The abundance of *Erysipelotrichaceae* in the gut microbiota of the LCB and MCB groups increased. *Erysipelotrichaceae* are butyrate-producing bacteria that efficiently degrade polysaccharides and fibers, producing short-chain fatty acids. Certain species within this family are associated with lipid metabolism (13).

#### Analysis of species structures at phylum and genus levels

**Fig 7:**
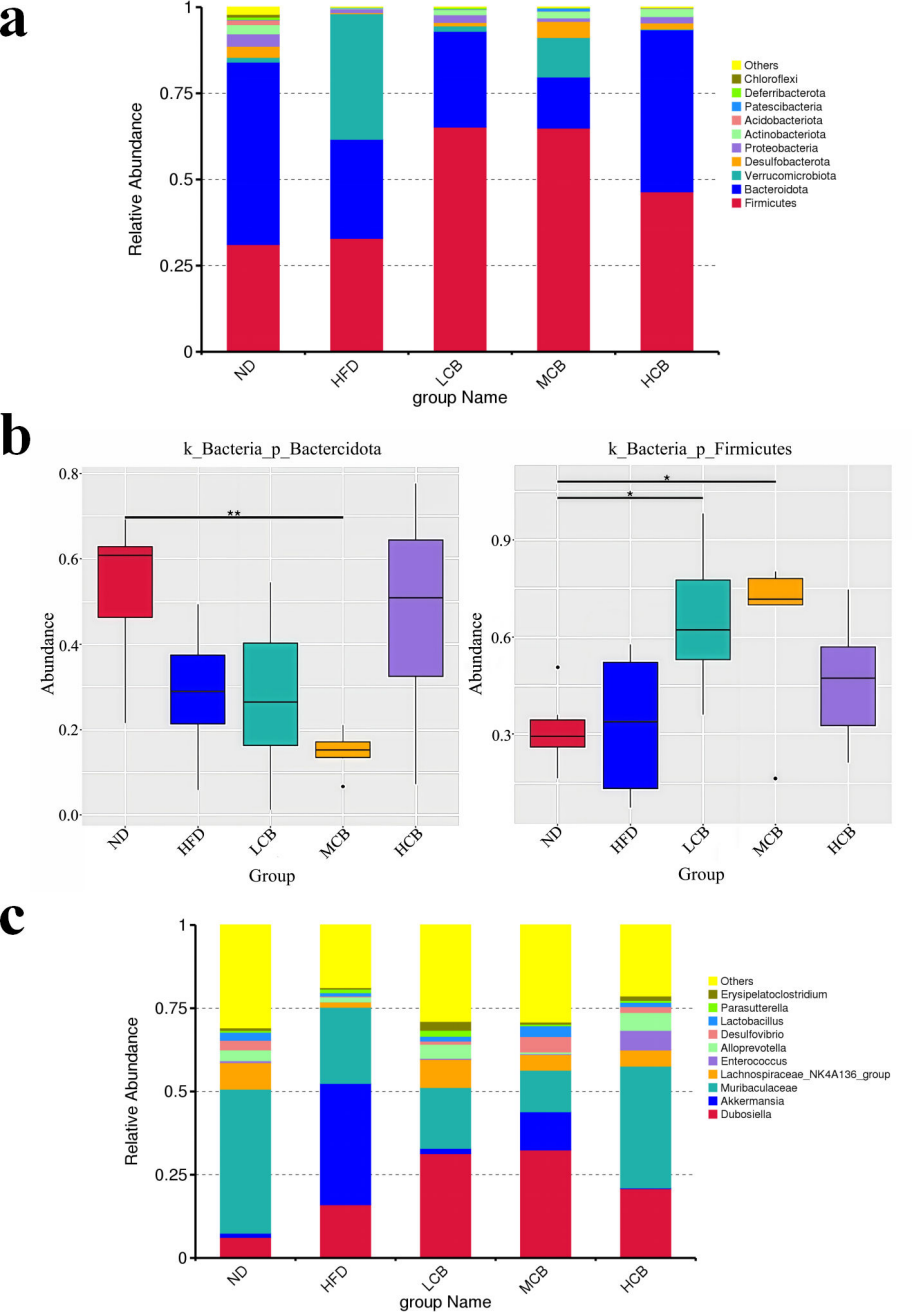
Analysis of species structure at the phylum level and the genus level (**a**, changes in the relative abundance of gut microbiota phylum; **b**, differences in species among the *Firmicutes* and *Bacteroidetes* groups at the phylum level; **c**, changes in the relative abundance of intestinal microbiota at the genus level) (ND, normal diet; HFD, high-fat diet; LCB, low dose *C. butyricum* B-3; MCB, medium dose *C. butyricum* B-3; HCB, high dose *C. butyricum* B-3).

At the phylum level (Fig. 7a through c), the dominant bacterial groups in the intestines of the mice were *Firmicutes*, *Bacteroidota*, *Verrucomicrobiota*, and *Desulfobacterota*. The highest proportions were observed in *Firmicutes* and *Bacteroidetes* (Fig. 7a). Except for the HFD group, which had a combined abundance of *Firmicutes* and *Bacteroidetes* at 61.68%, the sums of these phyla in the other groups exceeded 75%. Ley et al. (49) reported that obese mice exhibit a decrease in *Bacteroidetes* and an increase in *Firmicutes* compared to normal mice. This trend was also observed in the HFD, LCB, and MCB groups, which showed a decrease in the abundance of *Bacteroidetes*. In comparison, the abundance of *Bacteroidetes* and *Firmicutes* in the HCB group was closest to that in the ND group (Fig. 7b), indicating that the high dose of *C. butyricum* B-3 mitigated the imbalance of intestinal flora induced by a high-fat diet.

At the genus level (Fig. 7c), compared to the ND group, the HFD group exhibited a decreased abundance of *Muribaculaceae*, *Trichomonas*, and *Lactobacillus*. This suggests that a high-fat diet contributes to a reduction in the prevalence of beneficial gut bacteria in mice. In contrast, compared to the HFD group, the HCB group demonstrated an increase in the abundance of *Lactobacillus*, *Muribaculaceae*, *Molluscum,* and *Dubosiella*, indicating that *C. butyricum* B-3 enhances the presence of beneficial gut bacteria in mice. *Lactobacillus* is extensively distributed throughout the intestinal tract. Ji et al. (50) reported that *Lactobacillus sakei* CJLS03 could strengthen intestinal barrier integrity, suppress pro-inflammatory cytokines (e.g., tumor necrosis factor-α [TNF-α] and interleukin-6 [IL-6]), and promote anti-inflammatory cytokines (e.g., interleukin-10 [IL-10]) through the metabolism of short-chain fatty acids (SCFAs). This activity mitigates obesity-associated chronic low-grade inflammation and adipose tissue dysfunction. Moreover, the abundance of *Lactobacillus* is inversely associated with obesity. Certain strains of *Lactobacillus* have been confirmed to ameliorate obesity induced by a high-fat diet (34). It has been observed that with an increased abundance of *Muribaculaceae*, body weight and fat mass in obese subjects are significantly reduced, illustrating a negative correlation between *Muribaculaceae* and the onset of obesity (49). Additionally, Zhu et al. (51) reported that the relative abundance of *Muribaculaceae* is inversely related to body weight, body mass index (BMI), adipose tissue mass, and insulin sensitivity. Consequently, the enhanced abundance of *Muribaculaceae* and *Lactobacillus* due to *C. butyricium* B-3 treatment could be a primary factor in preventing fat accumulation.

The aforementioned results demonstrate that a high dose of *C. butyricum* B-3 can ameliorate the imbalance in gut microbiota caused by a high-fat diet. The colonization and persistence of strains in the gut contribute to these beneficial effects. Pan et al. (52) found that the probiotic strain *C. butyricum* CB2 exhibited strong adhesion to intestinal epithelial cells. Furthermore, Luo et al. (53) reported that *C. butyricum* predominantly colonizes the colon, and following gavage for seven consecutive days, it was retained in the intestinal tract of mice for at least 6 days. These findings provide a foundation for investigating the probiotic colonization capacity and the enhancement of microflora for preventing gastrointestinal diseases. Future studies should further analyze the colonization abilities of *C. butyricum* B-3 in the intestinal tract.

#### Analysis of short-chain fatty acids in mice on a high-fat diet

Short-chain fatty acids (SCFAs) are metabolic byproducts produced by the intestinal microbiota and are pivotal in reducing the pH within the intestinal tract. These SCFAs include acetic acid, propionic acid, butyric acid, valeric acid, isobutyric acid, and isovaleric acid. Notably, acetic acid, propionic acid, and butyric acid together constitute over 90% of the total SCFA content. SCFAs are essential for maintaining host health and regulating lipid metabolism and immune functions.

**Fig 8:**
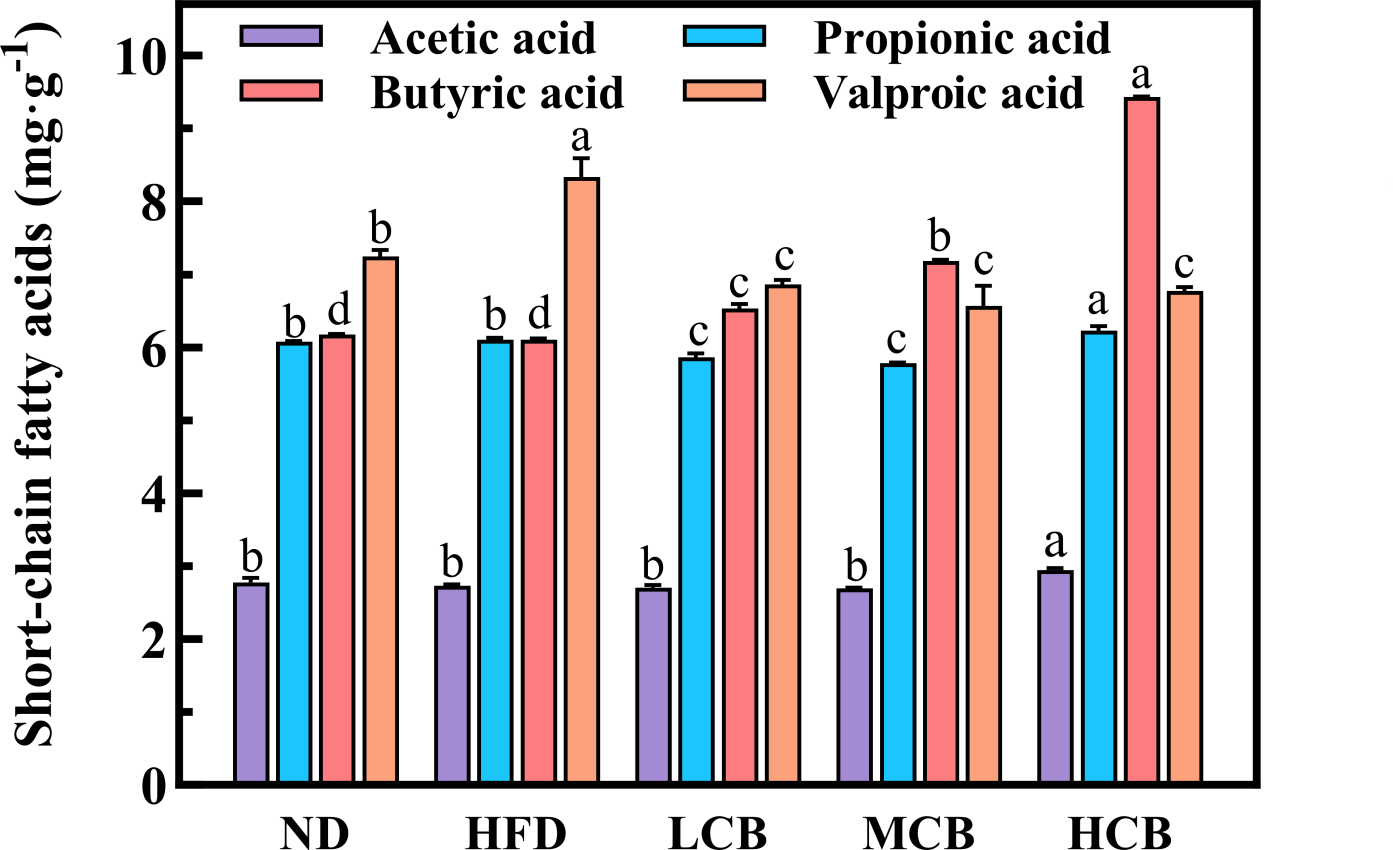
Analysis of short-chain fatty acids in mouse gut.

The concentrations of SCFAs in the fecal samples from the colons of mice were reported in Fig. 8. Notably, the concentration of valeric acid in the HFD group was significantly higher than that in the ND group (*P* < 0.05). In contrast, the concentrations of acetic acid, propionic acid, and butyric acid showed no significant differences between the two groups. Previous research suggests that high-fat diets, rich in specific fatty acids, may promote the production of valeric acid within the intestinal tract (54).

Among the experimental groups, the HCB group demonstrated the highest levels of acetic acid, propionic acid, and butyric acid. This indicates that a high dose of *C. butyricum* B-3 can enhance the metabolic capabilities of the intestinal microbiota, thereby facilitating an increased production of SCFAs. These acids perform various physiological regulatory functions, such as providing energy for colonic epithelial cells, strengthening the intestinal barrier, and preventing pathogen invasion (55). Specifically, *C. butyricum* B-3 significantly increased the levels of acetic acid, propionic acid, and butyric acid, with a notable increase in butyric acid. According to “Analysis of species structures at phylum and genus levels,” above, the HCB group also showed an increased abundance of beneficial bacteria, including *Muribaculaceae* and *Dubosiella. Muribaculaceae* can produce butyric acid by metabolizing specific amino acids (AAs), namely, threonine, aspartic acid, glycine, glutamic acid, and alanine (56). Additionally, *Dubosiella newyorkensis* has been shown to produce SCFAs, particularly propionic acid and L-lysine (57).

Acetic acid is pivotal in maintaining the acid-base balance in the intestine, fostering the proliferation of beneficial bacteria, and enhancing both blood flow and oxygen absorption in the colon (55). Propionic acid, on the other hand, functions to suppress appetite, reduce cholesterol levels, and inhibit fat storage. It also serves as a signaling molecule, orchestrating the immune functions of both the intestinal system and the broader body by engaging specific receptors (58). Additionally, butyric acid is identified as a key regulator of tight junction proteins (TJPs), with studies indicating its role in bolstering intestinal barrier function through the upregulation of zonula occludens-1 (ZO-1) and the redistribution of Occludin (59). Consequently, the administration of *C. butyricum* has been shown to significantly enhance intestinal health in mice subjected to high-fat diets.

Moreover, the strain *C. butyricum* B-3 is noted for its high butyric acid production. This short-chain fatty acid critically influences the metabolic and inflammatory markers in mice. Oral administration of *C. butyricum* B-3 regulates the gut flora and stimulates the production of other short-chain fatty acids, thereby modulating lipid metabolism. This intervention also optimizes the microbiota structure, encouraging the growth of other beneficial bacteria such as *Erysipelotrichaceae* and *Lactobacillus*. Consequently, the improved intestinal microbiota contributes to better weight management and lipid metabolism in mice on high-fat diets, thereby reinforcing intestinal health.

### Conclusion

Butyric acid exhibits diverse functions that enhance physical health. The study focused on the *C. butyricium* B-3 strain, which is characterized by its robust production of butyric acid. This strain demonstrated significant antioxidant capacity and lipid-lowering properties *in vitro*, alongside notable anti-obesity effects in a high-fat diet mouse model. Specifically, various dosages—low (10^6^ CFU/day), medium (10^7^ CFU/day), and high (10^8^ CFU/day)—of *C. butyricium* B-3 were effective in mitigating excessive weight gain induced by a high-fat diet. Furthermore, improvements were observed in lipid metabolism indicators, with the medium dose yielding the most substantial reduction in inflammatory factors. The high dose was particularly effective in enhancing gut microbiota and boosting the production of short-chain fatty acids. Therefore, this strain holds promise for development as a functional food aimed at combating obesity. Nonetheless, the potential of *C. butyricum* for further exploration in obesity research remains vast. At the mechanistic level, the specific molecular pathways and mechanisms require deeper investigation. Additionally, the translation of findings from *in vitro* studies and animal experiments into clinical applications needs to be progressively pursued.

## Data Availability

The sequencing data are stored in GenBank with the accession number PX353565.
